# Ginseng, Tribulus Extracts and Pollen Grains Supplementation Improves Sexual State, Testes Redox Status, and Testicular Histology in Nile Tilapia Males

**DOI:** 10.3390/antiox11050875

**Published:** 2022-04-29

**Authors:** Abdallah Tageldein Mansour, Ahmed Saud Alsaqufi, Eglal Ali Omar, Hossam S. El-Beltagi, Tarek Mohamed Srour, Mokhtar Ibrahim Yousef

**Affiliations:** 1 Al Bilad Bank Scholarly Chair for Food Security in Saudi Arabia, The Deanship of Scientific Research, The Vice Presidency for Graduate Studies and Scientific Research, King Faisal University, Al-Ahsa 31982, Saudi Arabia; aalsaqufi@kfu.edu.sa (A.S.A.); helbeltagi@kfu.edu.sa (H.S.E.-B.); 2 Department of Aquaculture and Animal Production, College of Agriculture and Food Sciences, King Faisal University, Al-Ahsa 31982, Saudi Arabia; 3 Department of Fish and Animal Production, Faculty of Agriculture (Saba Basha), Alexandria University, Alexandria 21531, Egypt; e.aomar@alexu.edu.eg (E.A.O.); tarek-srour@alexu.edu.eg (T.M.S.); 4 Agricultural Biotechnology Department, College of Agriculture and Food Sciences, King Faisal University, Al-Ahsa 31982, Saudi Arabia; 5 Biochemistry Department, Faculty of Agriculture, Cairo University, Giza 12613, Egypt; 6 Department of Environmental Studies, Institute of Graduate Studies and Research, Alexandria University, Alexandria 21526, Egypt; yousefmokhtar@alexu.edu.eg

**Keywords:** antioxidant status, sexual performance, Nile tilapia, *Tribulus terrestris*, ginseng, date palm pollen

## Abstract

This study aimed to investigate the effect of dietary supplementation of three natural antioxidants on sex hormone levels, enzymatic and non-enzymatic antioxidant systems, and histological changes in the testes of male Nile tilapia, *Oreochromis niloticus*. A total of 210 male Nile tilapia were distributed into seven treatments (three replicates for each) with an initial weight of 3.67 g fish^−1^. The fish were fed experimental diets (32% crude protein) without supplementation as control or supplemented with ginseng extract (GE; 0.2 and 0.4 g GE kg^−1^ diet), *Tribulus terrestris* extract (TT; 0.6 and 1.2 g TT kg^−1^ diet), and date palm pollen grains (DPPG; 3 and 6 g DPPG kg^−1^ diet) for 84 days. The results revealed a significant increase in the luteinizing hormone level with TT, DPPG, and GE supplementation increased the levels by 22.9%, 18.5%, and 17.6%, respectively. The testosterone level also increased significantly with TT_1.2_, GE_0.4_, TT_0.6_, and DPPG_6_ by 86.23%, 64.49%, 57.40%, and 24.62%, respectively. The antioxidant status in the testis homogenate showed a significant decrease in the level of thiobarbituric acid-reactive substances when using different dietary substances. In addition, glutathione reduced contents, glutathione *S*-transferases, glutathione peroxidase, catalase, and superoxide dismutase activities significantly increased with different dietary supplementation in a dose-dependent manner. The histological evaluation revealed normal histological features of the testes in all treatments with increasing active seminiferous tubules (%) in GE, TT, and DPPG supplemented groups, especially with the highest levels. In conclusion, the dietary supplementation of GE, TT, and DPPG enhanced sex hormones level, redox status, and testis structure and could improve the male reproductive performance of Nile tilapia.

## 1. Introduction

The male reproductive function is highly affected by free radical-induced oxidative stress, whereas the rapid replication of spermatogonia and the high rate of mitochondrial oxygen consumption in the testis, which is associated with a high level of unsaturated fatty acids, causing it to be very sensitive to oxidative damage [1,2]. Oxidative stress can contribute to the impairment of spermatogenesis, leading to male infertility [3]. In addition, the resistance of the spermatozoa to the effect of oxidative damage is lower than the other germ cells due to the lower enzymatic antioxidant system associated with reduced spermatozoa cytoplasm [4]. Therefore, the antioxidant system in the testis alone is not able to mitigate the oxidative stress complications; accordingly, antioxidant supplementation is necessary to contribute with the endogenous enzymatic antioxidants for preventing oxidative stress and maintaining proper oxidant/antioxidant balance [2,5]. Recently, the use of medicinal plants to enhance male functions in aquaculture is now receiving more attention for their antioxidant properties or for sexual hormone stimulation [6,7,8]. A massive number of herbs and plant extracts are available for inclusion in animal diets [9,10].

Ginseng is one of the most famous medicinal herbs worldwide; it has been used in traditional Chinese medicine since antiquity [11]. Ginseng is a rich source of phytochemicals, including ginsenosides, nitrogenous substances, carbohydrates, phytosterol, organic acids, essential oils, amino acids, and peptidoglycans [11,12]. *Panex ginseng* is the most commercially available species; it has been used as a growth promoter, immune stimulator, anti-inflammatory, antidiabetic, antitumor, anti-obesity, cardioprotective, antimicrobial, neuroprotective, aphrodisiac, and antioxidant [13,14]. The effect of ginseng extracts (GE) on male reproductive function was recently reported in rainbow trout (*Oncorhynchus mykiss*), and the results revealed an improvement in both semen quality and fertilization rate after increasing ginseng supplementation levels [6].

*Tribulus terrestris* (TT) is an annual shrub that belongs to the family Zygophyllaceae. This plant grows well in subtropical climates and deserts in different regions around the world [15]. The majority of chemical constituents within the seeds are steroidal saponins, referred to as protodioscin, containing flavonoids, flavonol glycosides, and alkaloids [15,16]. The medicinal use of this plant includes aphrodisiac, hypolipidemic, astringent, stomachic, immunomodulatory, antihypertensive, diuretic, hepatoprotective, and antimicrobial [17,18]. The TT has been used in aquaculture as a sex-reversal inducer in many fish species, and it has also been used to improve reproductive performance [8,19] Gharaei, et al. [20].

The date palm (*Phoenix dactylifera* L.) tree is one of the most cultivated trees in the middle east region and several hot and arid climate regions worldwide. The date palm belongs to the family Arecaceae, with male and female flowers occurring on separate plants [21]. The male gametes (date palm pollen grains; DPPG) are a fine powder that is used for transferring pollination. The DPPG contains an interesting macro and micro nutritional content, including vitamins, mineral salts, sugars, lipids, growth factors, enzymes, and co-factors [22]. The DPPG is a rich source of phenolic and flavonoid contents and antioxidant compounds [23]. It also contains hormone and hormone-like substances, including estrone, a-amirin, triterpenoidal saponins, and crude gonadotrophic substances [24,25]. Accordingly, DPPG has antioxidant [23], antibacterial, antiviral [26], anti-inflammatory [27], aphrodisiac [28], and hepato-protective properties [29]. In our previous study, the dietary supplementation of GE, TT, and DPPG revealed an improved growth performance and feed utilization of Nile tilapia [30]. Meanwhile, the use of GE, TT, and DPPG in improving the reproductive function of Nile tilapia is rare. Therefore, the present study aimed to evaluate the effect of GE, TT, and DPPG in different concentrations on the sexual hormone secretion, enzymatic and non-enzymatic antioxidants, and the histological changes in the testis of Nile tilapia, *Oreochromis niloticus*.

## 2. Materials and Methods

### 2.1. Fish and Experimental Facilities

A sample of 210 healthy male Nile tilapia, *O. niloticus* fingerlings were manually separated, with an average initial body weight of 3.67 ± 0.02 g fish^−1^ obtained from a private fish farm (Kafr–El Sheikh Governorate, Egypt). The fish were kept for two weeks in indoor circular fiberglass tanks (1 m^3^) as an acclimation period and fed on a control diet prior to the start of the experiment. Twenty-one glass aquaria with dimensions of 100 × 30 × 40 cm and a 100 L volume of water for the aquaria were used. The water temperature averaged 26 ± 2 °C. Continuous aeration is maintained in each aquarium using an electric air blower. A manual method for the removal of excreta was conducted every day before the first feeding by replacing half of the water volume with an equal volume of fresh water to maintain acceptable water quality.

### 2.2. Experimental Design

Seven treatments were applied in three replicates, each stocked with ten fish. The following treatments were used: control fed basal diets without any supplementations, GE_0.2_ (supplemented with 0.2 g GE kg^−1^ diet), GE_0.4_ (supplemented with 0.4 g GE kg^−1^ diet), TE_0.6_ (supplemented with 0.6 g TT kg^−1^ diet), TT_1.2_ (supplemented with 1.2 g TT kg^−1^ diet), DPPG_3_ (supplemented with 3 g DPPG kg^−1^ diet), and DPPG_6_ (supplemented with 6 g DPPG kg^−1^ diet). The tested levels were selected according to the literature data [22,31,32]. The experiment lasted 84 days. The study was carried out in accordance with the Declaration of Helsinki’s standards and was authorized by Alexandria University’s Institutional Animal Care and Use Committee with approval No. (AU:19/21/06/25/3/22).

### 2.3. Experimental Diets

The experimental diets (32% crude protein) were prepared in the laboratory using a small mincer (3.0 mm diameter), dried in a forced-air oven (40–50 °C), packed in plastic pages, and stored in the refrigerator (4 °C). All feed ingredients were purchased from the local market (Table 1) and finely ground, mixed well then incorporated into the diet. The supplementations (GE, TT, and DPPG) were added to the diets by excluding the same portion of wheat flour. The phytochemicals used were obtained or prepared as follows: Ginseng extract, *P. ginseng* (Ginsana^®^; contains G115 with 4% ginsenosides), was kindly supplied via the Pharco-Pharmaceuticals Co., Alexandria, Egypt. *Tribulus terrestris* extract, *T. terrestris* (Trib Gold^®^; 40% protodioscin), was purchased from the Nerhadou International Co. for Pharmaceuticals and Nutraceuticals, 6th of October City, Egypt. Date palm, *P. dactylifera*, pollen: Fresh pollen from date palms was collected from Edku city, Egypt. The pollen grains were separated from the kernels with a fine gauze sieve and oven-dried at 40–50 °C for 24 h. All supplementations were weighed and mixed carefully into the oil, then added to the basal diet with each respective level.

Fish in each aquarium were hand-fed two times a day at 9.00 a.m. and 14.00 p.m. at a rate of 6% in the first four weeks, 5% in the second four weeks, and 4% until the end of the experimental period (84 days). The feeding rates were adjusted according to the weekly change in live body weights [33].

### 2.4. Samples Collection

At the end of the experiment, blood samples were collected from the caudal vein of anesthetized fish (50 mg clove oil L^−1^) using a sterile 1-mL syringe containing 50 μL of heparin (IU mL^−1^, Amoun Pharmaceutical Co. S.A.E., El Obour city—Cairo, Egypt). Blood samples were taken from 12 fish per treatment (four fish tank^−1^) and centrifuged (1075× *g*, 10 min, 4 °C) to obtain plasma samples, which were stored at −80 °C until the use in the biochemical assays. The same fish used for blood sampling were dissected, and testes were separated, weighed, and divided into portions. The first portion was subjected to a histological procedure. The second portion was minced and homogenized (10% *w*/*v*) in ice-cold sucrose buffer (0.25 M) in a Wise Tis^®^ HG-15D homogenizer (Daihan Scientific, Bangalore, India). The homogenate was centrifuged at 7063× *g* for 20 min at 4 °C. The resulting supernatant was collected and stored at −80 °C.

### 2.5. Measured Parameters

#### 2.5.1. Testes Somatic Index

The testes’ somatic index (%) was calculated as g/100 g body weight with the following formula:

Teste’s somatic index = 100 × [Testes weight (g)/body weight (g)].(1)

#### 2.5.2. Sexual Hormones

- Luteinizing hormone determination

Stored plasma samples were analyzed for luteinizing hormone (LH; IU/L) by an Automated Enzyme Immunoassay system called the immulite/immulite 1000 system (AIA-360; Tosoh India Pvt. Ltd., Goregaon (East) Mumbai, India), which is based on the methods described by Beitins et al. [34].

- Testosterone determination

The stored plasma samples were analyzed for testosterone (ng mL^−1^) by the enzyme-linked immune sorbent assay (ELISA kits; DRG instrument GmbH, Marburg, Germany) according to Abraham [35]. The method is based on competitive binding, where an unknown amount of testosterone in the sample and a defined amount of testosterone conjugated to horseradish peroxidase compete for the binding sites of testosterone antiserum coated to the well of the microplate. After one hour of incubation in a shaker, the microplate was washed four times, then the substrate was added, and the reaction was stopped by 2N hydrochloric acids. The concentration of testosterone is inversely proportional to the optical density at 450 nm.

#### 2.5.3. Antioxidant Status Assays

- The glutathione *S*-transferases

The glutathione *S*-transferases (GST; EC 2.5.1.18) were determined by adding 12.5 µL of testes homogenate with 682.5 µL potassium phosphate buffer (pH 6.5), 50 µL reduced glutathione (5 mM), and 5 µL P-Nitrobenzyl chloride (1 mM L^−1^) and mixed by vortex and incubated for 20 min at room temperature. The absorbance was read at 310 nm by a UV-double beam spectrophotometer [36].

GST enzyme activity in tissue (µmol/h) = (Optical density/∆e) × 3(2)

where: ∆e (the difference in the molar extinction coefficient) = 1.9 mM^−1^ cm^−1^.

- Glutathione peroxidase

Glutathione peroxidase (GSH-Px; U mg^−1^ protein; EC 1.11.1.9) activity was assayed using the method of Flohé and Günzler [37] in tissue homogenate. Briefly, 25 µL of testes homogenate was incubated for 5 min at 37 °C with 375 µL Tris HCl buffer (pH 7.6, 0.05 M), 50 µL reduced glutathione (pH 7.6), and 50 µL cumene hydroperoxide (pH 7.6). Then, 0.5 mL trichloroacetic acid (15%) was added to stop the reaction, vortexed, incubated for 10 min, and centrifuged (7063× *g* for 10 min at 4 °C). The supernatant (250 µL) was transferred to 0.5 mL Tris HCl buffer (pH 8.9) and 25 µL of 5,5′-dithiobis (2-nitrobenzoic acid; DTNB) was added. The absorbance was read at 412 nm (Spectrophotometer PD-303 UV, APEL, Saitama, Japan). A control tube was prepared for each sample by adding cumene after adding trichloroacetic acid.

GSH-Px specific enzyme activity (IU/g wet tissue) = 25 × (E × 6.2 × 10/13.1 × 0.05)(3)

where E: difference in absorbance between control and sample; 6.2: extinction coefficient of cummen hydroperoxide; 13.1: extinction coefficient of DTNB; 0.05: volume of sample; 10: dilution coefficient.

- Catalase

Catalase activity (CAT; U mg^−1^ protein; EC 1.11.1.6) was measured according to Luck [38]. Briefly, a 10 µL tissue homogenate sample was added to 1.25 µL of freshly prepared buffer containing 50 mL of H_2_O^2^ 10 mL^−1^ Na-K-phosphate buffer (0.15 M, pH 7, El-Gomhoria Co., Cairo, Egypt). The difference in absorbance was recorded after 20 s (A1) and after 80 s (A2) of incubation at 240 nm against air. The CAT activity was calculated as A1-A2/0.0008.

- Superoxide dismutase

Superoxide dismutase (SOD; U mg^−1^ protein; EC 1.15.1.1) activity was evaluated according to Misra and Fridovich [39]. Briefly, 20 µL of tissue homogenate was added to 940 µL sodium carbonates buffer (pH 10.2, 0.05 M, El-Gomhoria Co., Egypt) and 40 µL epinephrine (30 mmol L^−1^ dissolved by adding 30 mL of HCL, Sigma, New York, NY, USA). The inhibition of epinephrine auto-oxidation in the alkaline medium to adrenochrome was recorded after 30 and 90 s at 480 nm. A control was prepared and consisted of 960 µL of sodium carbonate buffer and 40 µL of epinephrine.

The percent of inhibition (%) = 100 − [(∆A control − ∆A sample/∆A control) − 100].(4)

SOD activity (U g^−1^ tisuue) = % inhibition × 3.75. (5)

- Reduced glutathione

The reduced glutathione (GSH) levels were determined after the incubation of 150 µL of testes homogenate with 150 µL of sulfosalicylic acid (4%) for 5 min on ice, then the mixture was centrifuged 7063× *g* [40]. A total of 66 µL of supernatant was transferred into a glass tube containing 66 µL DTNB (Ellman reagent; 0.01 M, 0.4%) and 865 µL of potassium phosphate buffer pH (7.4). The absorbance was read after 5 min at 412 nm against sulfosalicylic acid (4%).

GSH concentration (µmol/g wet tissue) = 10 × (sample absorbance/0.049)(6)

- Thiobarbituric acid-reactive substances

The thiobarbituric acid-reactive substances (TBARs) were measured in homogenates at 532 nm using 2-thiobarbituric acid (TBA; 2-thioxodihydropyrimidine-4,6). The principle of the procedure is that, at low pH and elevated temperature, malondialdehyde (MDA; the end product of lipid peroxidation) readily participates in a nucleophilic addition reaction with TBA to form MDA:TBA adducts (1:2), generating red fluorescence. The color intensity is proportionate to MDA levels. An extinction coefficient of 156,000 M^−1^ cm^−1^ was used for the calculation [41]. TBARs (nmol/g tissue) = Sample Absorbance/156,000.

#### 2.5.4. Histological Examination

A small piece of the testes was removed from the experimental fish and rapidly placed in an adequate amount of 10% neutral buffered formalin for at least 24 h. The fixed specimens were processed through the conventional paraffin embedding technique (dehydration through ascending grades of ethanol, clearing in chloroform, and finally embedding in melted paraffin wax at 60 °C). Paraffin blocks were sectioned into 5 microns-thick sections stained with Hematoxylin and Eosin (H&E) according to the method described by Culling [42]. The stained sections were investigated using a light microscope at 100× magnification (B-293 LD 1.50, Optika, Via Rigla, Ponteranica, Italy). The percentage of active seminiferous tubules (ST) was measured by the determination of the number of ST containing a considerable amount of spermatids/100 ST at a power of 100× in five consecutive sections.

### 2.6. Statistical Analysis

The results are expressed as the mean ± standard error (SE). All data were tested for homogeneity by Levene’s tests, and the normal distribution data were checked by Shapiro–Wilk. The normally distributed data were treated using one-way ANOVA by SPSS (Standard Version 17.0 SPSS Inc. Chicago, IL, USA). Duncan’s multiple range test was used to compare the differences between means when significant F values were observed at the *p* ≤ 0.05 level [43]. Non-normally distributed data were analysed by the non-parametric Kruskal–Wallis test followed by multiple comparison tests. In addition, the difference between the effect of two doses of each supplementation was compared by paired sample *t*-tests and presented in figures. Arcsine transformation was used for the statistical analysis of active seminiferous tubules (%) using one-way ANOVA.

## 3. Results

### 3.1. Sexual Hormones and Testis Somatic Index

**Figure 1:**
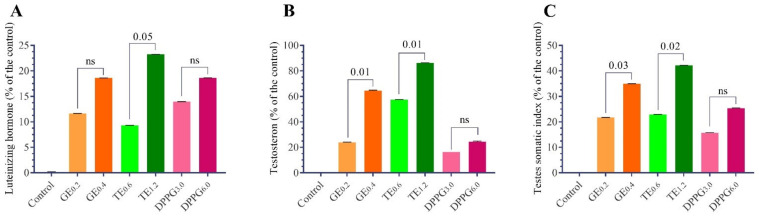
Effect of different antioxidants supplementation (g/kg diet) on changes of luteinizing hormone (**A**), testosterone levels (**B**) and testis somatic index (**C**) as a percent of the control of male Nile tilapia, *O. niloticus*. GE, ginseng extract; TT, *Tribulus terrestris* extract; DPPG, date palm pollen grains. (n = 15) ns: non significant, 0.05–0.001 the *t*-test significant levels of actual parameter values.

The results presented in Table 2 summarize the effects of antioxidant supplementation (GE, TT, and DPPG) on LH and testosterone levels in the plasma of Nile tilapia, *O. niloticus*, fingerlings. The results indicated that the fish which received diets containing all dietary supplementations had a significant increase in LH hormone levels compared to the control, and the effects were level-dependent on each supplementation (Figure 1). The highest LH levels were recorded with TT (1.2 g/kg diet) followed by DPPG (6 g/kg diet) and GE (0.4 g/kg diet), and the percentage of increases were 22.9%, 18.5%, and 17.6%, respectively.

Plasma testosterone levels significantly increased with different supplementation treatments compared to the control. The most effective doses for testosterone levels were TE (1.2 g/kg diet) followed by GE (0.4 g/kg diet), TT (0.6 g/kg diet), and DPPG (6 g/kg diet), the percent of increase reached 86.23%, 64.49%, 57.40%, and 24.62%, respectively, more than the control (Table 2 and Figure 1). The testis somatic index increased significantly with higher doses of different supplementations compared to the control (Table 2 and Figure 1).

### 3.2. Antioxidant Status

**Figure 2:**
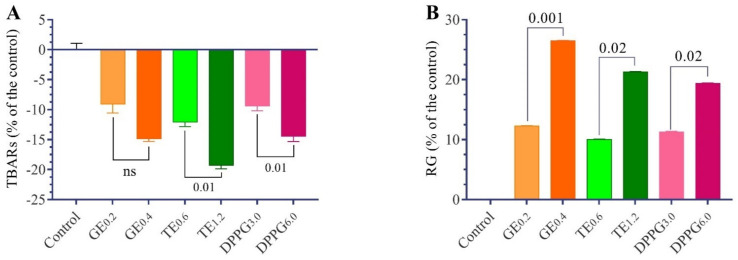
Effect of different antioxidants supplementation (g/kg diet) on changes of thiobarbituric reactive substances (TBARs; (**A**)) and reduced glutathione (RG; (**B**)) contents as a percent of the control in testes homogenate of male Nile tilapia, *O. niloticus*. GE, ginseng extract; TT, *Tribulus terrestris* extract; DPPG, date palm pollen grains. (n = 15) ns: non-significant, 0.05–0.001 the *t*-test significant levels of actual parameter values.

**Figure 3:**
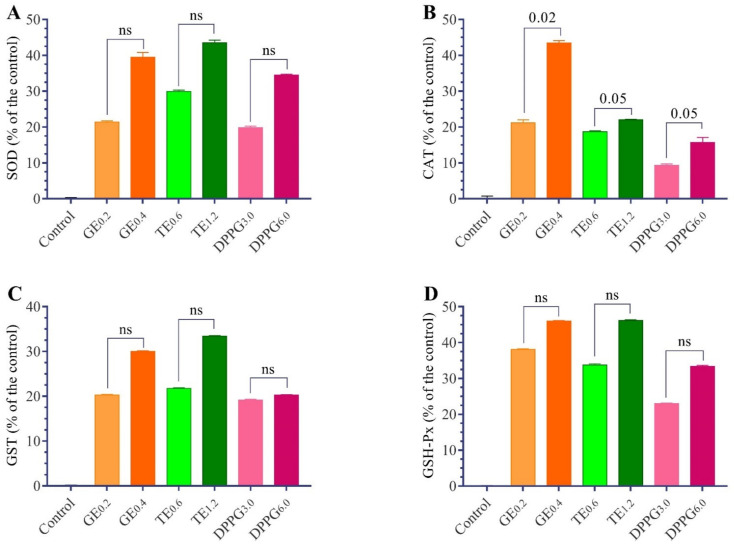
Effect of different antioxidants supplementation (g/kg diet) on changes of superoxide dismutase (SOD; (**A**)); catalase (CAT; (**B**)); glutathione-S-transferase (GST; (**C**)) and glutathione peroxidase (GSH-Px; (**D**)) activities as a percent of the control in testes homogenate of Nile tilapia, *O. niloticus*. GE, ginseng extract; TT, *Tribulus terrestris* extract; DPPG, date palm pollen grains. (n = 15) ns: non-significant, 0.05–0.001 indicates *t*-test significant levels of actual parameter values.

The changes in antioxidant status in the testes of Nile tilapia fed with different antioxidant supplementations are presented in Table 3 and Figure 2 and Figure 3. The TBARs levels decreased significantly in all dietary supplements of GE, TT, and DPPG. Meanwhile, the reduced glutathione content and the increased activities of CAT, SOD, GST, and GSH-Px were significantly different with different supplementation in a dose-dependent manner concerning the supplementations. The GE has the highest antioxidant properties, followed by TT. The *t*-test analysis revealed that the changes between the respective level of each supplementation were not significant except with RG, TBARs, and CAT.

### 3.3. Histological Examination

**Figure 4:**
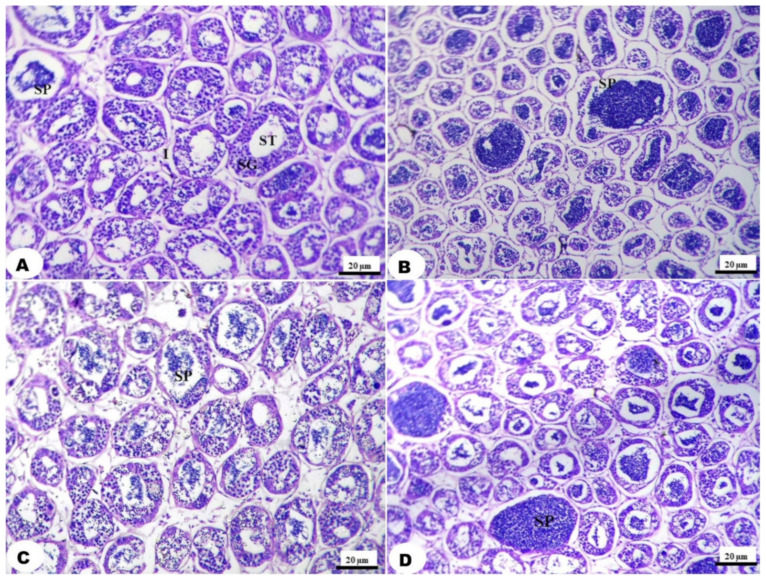
Testicular sections of Nile tilapia, O. *niloticus*, fingerlings, stained with haematoxylin and eosin stain: (**A**) Control group showing normal histology featuring seminiferous tubules (ST) lined by multilayer of spermatogonia (SG) and separated by thin strands of interstitial connective tissue contain leydig cells (I), some ST are active contain considerable amount of spermatids (SP) in its lumen. (**B**) GE_0.4_ group showing normal histology with 37.45% active ST (SP). (**C**) TT _1.2_ group showing normal histology with 27.95% active ST (SP). (**D**) DPPG_6_ showing normal histology with 33.45% active ST (SP).

**Figure 5:**
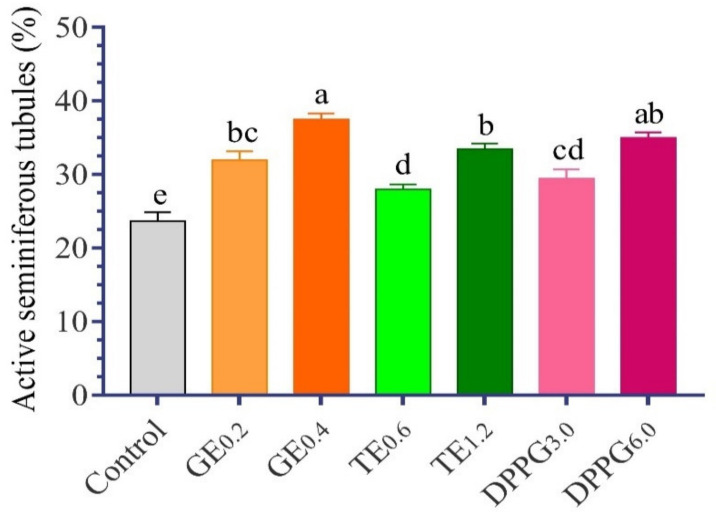
Effect of different antioxidants supplementation (g/kg diet) on testies percentage of active seminiferous tubules (%) of of Nile tilapia, *O. niloticus*. GE, ginseng extract; TT, *Tribulus terrestris* extract; DPPG, date palm pollen grains. (n = 5). Column superscripted by different alphabets within the same column are significantly different (*p* < 0.05).

The histological examination of the testis showed a nearly normal histological appearance in the control group (Figure 4 and Figure 5). Figure 4A shows a histological section of the control group with normal histology featuring seminiferous tubules (ST) lined by a multilayer of spermatogonia and separated by thin strands of interstitial connective tissue containing Leydig cells, with low active ST (23.64%) and actively containing a considerable number of spermatids. All dietary supplementations showed a normal histological appearance and a slight to high improvement in spermatids abundance. Whereas the treatment with GE at the low level showed 31.89% active ST. Meanwhile, the abundance of spermatids in testis fed with a high GE supplemented diet had the highest active ST, with 37.45% (Figure 4B). In addition, the treatment with TT showed a moderate abundance of spermatids in the lumen of ST in a level-dependent manner of 27.95 and 33.45%, respectively (Figure 4C and Figure 5). The treatment with DPPG at both levels showed an increased abundance of spermatids in the lumen of the testicular tubules with increasing supplementation levels, as active ST was 29.39 and 34.92%, respectively (Figure 4D and Figure 5).

## 4. Discussion

In the testes, the continuous replication of spermatocytes and production of sperms results in an expected occurrence of oxidative stress. However, the excessive production of reactive oxygen species associated with the high rate of metabolic processes in the testes could be ameliorated by endogenous antioxidant enzymes and exogenous dietary antioxidants [5,44]. The dietary antioxidant can delay lipid peroxidation by inhibiting the initiation or propagation phase of oxidizing chain reactions by scavenging free radicals [45]. Accordingly, the present study examined the effect of dietary supplementation of different natural antioxidants (GE, TT, and DPPG) on the enzymatic and non-enzymatic antioxidant system, gonadosomatic axes, and the teste histological structure of Nile tilapia, *O. niloticus*. The experiment continued for 84 days before the final weights were achieved (38.64–49.05 g fish^−1^); at this weight, the males of Nile tilapia can reach the first maturation size [46].

The supplementation with GE, TT, and DPPG improved the enzymatic (GSH-Px, GST, CAT, and SOD activities) and non-enzymatic antioxidant markers (GSH content and TBARs levels) in the testes homogenate of treated Nile tilapia males. In accordance with the obtained results, dietary ginseng decreased lipid peroxidation and improved SOD, GSH-Px, GST, GR, and CAT activities and GSH, ascorbic acid, and α-tocopherol contents in rats testes [47,48]. Moreover, hybrid grouper fed with an increasing level of GE improved the activities of hepatic SOD and the total antioxidant capacity and expression of CAT and GR in a dose-dependent manner [49]. The improvement of antioxidant enzyme activities in the present study with GE supplementation could be attributed to the presence of ginsenosides which regulated the pathways of extracellular-signal-regulated kinase and mitogen-activated protein kinase (p38), which in turn improved the expression of different antioxidant enzymes in the testes [50]. In addition, the dietary TT showed an improvement in antioxidant defense systems in different animals, including *O. mossambicus* [19,51], piglets [52], and chickens [53]. Moreover, the dietary supplementation of TT improved testicular enzyme activities in *Poecilia latipinna* fish [19]. The phenolic content of TT is the main antioxidant agent in the fruit extracts [54].

Moreover, the improvement of antioxidant balance in the testes homogenate of fish fed DPPG supplemented diet in the present study agreed with the increase in testicular antioxidants the glutathione content and GST, GSH-Px, and SOD activities in diabetic rats [55]. The dietary DPPG alleviated the oxidative stress in the testis of cadmium chloride-exposed animals [56,57] and diabetic rats [55]. DPPG could be considered a promising source of new natural antioxidants, including flavonoids and phenolic compounds [23]. It has antioxidant activities, such as radical scavenging activity and inhibiting lipid peroxidation [58].

The present findings proved that GE, TT, and DPPG improved the antioxidant status in the testes of Nile tilapia, *O. niloticus*, by increasing the activities of the enzymatic antioxidants and the content of non-enzymatic antioxidants and decreasing the level of free radicals. This improvement of antioxidant status in the testes of fish could positively improve the reproductive performance of the animals. However, fish spermatozoa are highly sensitive to oxidative stress-related damage, mainly due to the high content of PUFA and low content of spermatozoa cytoplasm [59]. Therefore, the spermatozoa cell wall, DNA, and protein are targets for oxidative stress. The determination of sperm quality is extremely difficult in fish; therefore, the search for quality estimators, including biochemicals investigation, is highly important [4]. Accordingly, improving the antioxidant balance in the testes of fish is an important measure for maintaining male reproductive performance.

In addition, the results of the present study revealed a significant increase in LH levels with the highest level of different dietary supplementations (GE, TT, and DPPG) by 17.56, 22.85, and 18.48%, respectively, compared to the control. In addition, the testosterone levels increased significantly with all supplementation levels, the percent of increase reached 64.49, 86.23, and 24.62%, with the highest levels of different supplementations compared to the control. These results were in agreement with Yun et al. [60], who reported that testosterone concentrations and sperm populations increased significantly with dietary supplementation of GE. In addition, male rainbow trout (*Oncorhynchus mykiss*) fed dietary supplementation of ginseng root extract improved sperm quality and reproductive performance [6].

The improvement of LH with dietary ginseng extract could be attributed to the direct stimulation of anterior pituitary gland cell functions, as shown by Tsai et al. [61], which in turn increased testosterone secretion [62]. Moreover, GE could regulate testosterone secretion by modulating gene expression in the testis, whereas it could up-regulate genes that are responsible for steroid hormone metabolism and the top molecular and cellular functions [63].

Concerning the elevation of testosterone levels in groups treated with TT, the current results were supported by the findings of Gharaei et al. [20], who found that testosterone and dehydroepiandrosterone were significantly increased with increasing TT extract supplementation in zebrafish, *Danio rerio*. In addition, the supplementation with TT significantly improved the level of testosterone, 11-keto testosterone, sperm concentration, and vitality in Nile tilapia males [8]. The effect of TT on testosterone levels may be directly attributed to the presence of protodioscin as the main active component, which is considered a testosterone a precursor [15]. Moreover, the indirect effect of TT is believed to affect testosterone levels by stimulating the release of LH, as confirmed in the present study, which serves to stimulate the natural production of testosterone [62].

The improving effect of date palm pollen on LH and testosterone was supported by Selmani et al. [64] and Iftikhar et al. [65], who revealed that dietary DPPG increased serum levels of LH and testosterone. Moreover, Abbass et al. [66] reported that honey bee pollen significantly increased testicular weight, gonadosomatic index, and improved the semen quality of male Nile tilapia, *O. niloticus*. Date palm pollen contains gonadotropic and steroidal compounds, which could participate in improving LH and testosterone secretion [25]. This will elucidate the increment of testosterone levels associated with DPPG supplementation. In addition, the increase in LH and testosterone secretion in the present study could be partially attributed to the antioxidant properties of the different used supplementations, as indicated in the improvement in the antioxidant balance in the present study (Figure 1 and Figure 2). These improvements could improve the function of different endocrine glands [8,61,64].

Furthermore, the increase in the testis’s somatic index in the current experiment is supported by the findings of Kavitha and Subramanian [19], who reported a significant increase in testis weight of *Poecilia latipinna* fish fed a TT extract-supplemented diet. Furthermore, Sharma et al. [67] reported that TT extract increased testes and epididymis weight in rats. Bahmanpour et al. [28] found an increase in the weight of testis and seminal vesicle in the rats that consumed date palm pollen suspension. In addition, ginseng produced a distinct testicular histological improvement in mercury-exposed animals [68]. The histological improvement in the present study could be due to the herbal contents of saponin, steroids, antioxidants, and different compounds. It has many effects, such as increased testosterone and dihydrotestosterone levels, stimulating sperm production [31,69], preventing cell death, and inhibiting the destruction of the mitochondrial membrane [70].

The testes histological investigation showed an increase in the abundance of spermatids in the lumen of the testicular lobules with different dietary supplementations. The best treatment was *T. trestriss* (1.2 g/kg diet) which showed a high abundance of spermatids. These results are supported by the findings of Cek et al. [71], who found that spermatogenesis was improved in TT treated group of *Cichlasoma nigrofasciatum* compared to the control group and the histological investigation of the testes in TT treated groups showed an increase in the number of spermatogenic cysts and excessive late stage spermatogenesis. In addition, the dietary supplementation of TT extract improved the reproductive performance of male convict cichlid, *C. nigrofasciatum* [72]. In addition, Abbass et al. [66] found that dietary bee pollen increased sperm accumulation and size of interstitial cells when compared to the control in Nile tilapia.

## 5. Conclusions

The dietary supplementation with GE, TT, and DPPG in the diets of male Nile tilapia, *O. niloticus*, enhanced the enzymatic and non-enzymatic antioxidants system in the testes. The sexual hormones level, such as the testosterone and luteinizing hormone levels, were improved with the highest supplementation levels of different antioxidant substances. The histological evaluation revealed normal histological features of the testes in all treatments with increasing active seminiferous tubules (%) in GE, TT, and DPPG supplemented groups, especially with the highest levels.

## Figures and Tables

**Table 1:** Ingredients and proximate composition (g kg^−1^ dry weight) of the experimental diet.

Ingredients	g kg^−1^
Fish meal (herring; 72%)	260
Corn gluten	150
Yellow corn	390
Rice bran	100
Wheat flour	50
Corn oil	24
Vitamins and Minerals primex ^1^	20
Calcium-mono phosphate	6
Proximate chemical composition	
Dry matter (DM)	922.00
Crude protein (CP)	323.80
Ether extract (EE)	72.00
Nitrogen-free extract (NFE) ^2^	489.80
Crude fiber (CF)	46.10
Ash	68.30
Gross energy (GE; kj g^−1^ DM) ^3^	18.90
P/E ratio (mg CP: kj) ^4^	17.13

^1^ Composition of vitamin mineral mixture of 1 kg: Vitamin A—50,00,000 IU; Vitamin D_3_—1,000,000 IU; Vitamin B_2_—2.0 g; Vitamin E—750 units; Vitamin K—1.0 g; Calcium pantothenate 2.5 g; Nicotinamide—10.0 g; Vitamin B_12_—6.0 g; Choline chloride: 150.0 g; Manganese sulphate anhydrous: 27.5 g; Potassium iodide: 1.0 g; Ferrous sulphate: 7.5 g; Zinc sulphate heptahydrate: 15.0 g; Copper sulphate: 2.0 g; Sodium selenite, 0.1 g; Cobalt chloride: 0.45 g; and calcium carbonate: up to 1000 g. ^2^ NFE: Nitrogen free extract calculated using the following equation: NFE = 100 − (crude protein + ether extract + crude fiber + ash). ^3^ GE: Gross energy calculated on the basis of 23.6, 39.4 and 17.2 k joule gross energy g^−1^ protein, ether extract and NFE, respectively. ^4^ P/E ratio: protein energy ratio (mg crude protein kj^−1^ gross energy) = CP/GE × 1000 [33].

**Table 2:** Effect of different levels of different antioxidant supplementation (g/kg diet) on luteinizing hormone, testosterone levels, and testis somatic index of Nile tilapia, *O. niloticus*, fingerlings.

Items	Luteinizing Hormone (IU/L)	Testosterone (ng/mL) [30]	Testes Somatic Index (%) [30]
Control	0.43 ± 0.02 ^b^	4.15 ± 0.13 ^d^	0.83 ± 0.03 ^c^
GE0.2	0.48 ± 0.03 ^a,b^	5.14 ± 0.26 ^c^	1.01 ± 0.03 ^a,b,c^
GE0.4	0.50 ± 0.02 ^a^	6.83 ± 0.30 ^b^	1.12 ± 0.04 ^a,b^
TT0.6	0.47 ± 0.02 ^a,b^	6.53 ± 0.18 ^b^	1.02 ± 0.04 ^a,b,c^
TT1.2	0.53 ± 0.01 ^a^	7.73 ± 0.16 ^a^	1.18 ± 0.05 ^a^
DPPG3.0	0.49 ± 0.01 ^a,b^	4.83 ± 0.03 ^c^	0.96 ± 0.10 ^b,c^
DPPG6.0	0.51 ± 0.03 ^a^	5.17 ± 0.28 ^c^	1.04 ± 0.12 ^a,b^

Values superscripted by different alphabets within the same column are significantly different (*p* < 0.05). GE, ginseng extract; TT, *Tribulus terrestris* extract; DPPG, date palm pollen grains. (n = 15) The testosterone level and testes somatic index cited in our previous paper [30].

**Table 3:** Effect of different antioxidants supplementation (g/kg diet) on the levels of thiobarbituric acid-reactive substances, reduced glutathione, and the activities of antioxidant enzymes in testes homogenate of Nile tilapia, *O. niloticus*, fingerlings.

Items	Thiobarbituric Acid-Reactive Substances (nmol/mL)	Reduced Glutathione (µmol/mL)	Superoxide Dismutase (U/mL)	Catalase (U/mL)	Glutathione *S*-Transferase (µmol/h)	Glutathione Peroxidase (U/mL)
Control	49.97 ± 1.05 ^a^	3.10 ± 0.09 ^d^	8.92 ± 0.33 ^b^	20.22 ± 0.79 ^d^	3.50 ± 0.18 ^b^	5.96 ± 0.14 ^c^
GE 0.2	45.44 ± 1.50 ^b^	3.48 ± 0.06 ^b,c^	10.83 ± 0.32 ^a,b^	24.50 ± 0.84 ^b^	4.21 ± 0.12 ^a^	8.23 ± 0.20 ^a^
GE 0.4	42.56 ± 0.47 ^b,c^	3.92 ± 0.08 ^a^	12.44 ± 1.37 ^a^	29.00 ± 0.68 ^a^	4.55 ± 0.16 ^a^	8.70 ± 0.19 ^a^
TT 0.6	43.94 ± 0.74 ^b^	3.41 ± 0.10 ^c^	11.59 ± 0.37 ^a^	24.00 ± 0.26 ^b,c^	4.26 ± 0.16 ^a^	7.97 ± 0.39 ^a,b^
TT 1.2	40.34 ± 0.59 ^c^	3.76 ± 0.07 ^a^	12.80 ± 0.74 ^a^	24.67 ± 0.15 ^b^	4.67 ± 0.13 ^a^	8.71 ± 0.25 ^a^
DPPG 3	45.29 ± 0.82 ^b^	3.45 ± 0.09 ^c^	10.69 ± 0.41 ^a,b^	22.11 ± 0.37 ^c,d^	4.17 ± 0.18 ^b^	7.33 ± 0.15 ^b^
DPPG 6	42.75 ± 0.86 ^b,c^	3.70 ± 0.08 ^a,b^	12.00 ± 0.24 ^a^	23.40 ± 1.36 ^b,c^	4.21 ± 0.07 ^a^	7.95 ± 0.31 ^a,b^

Values superscripted by different alphabets within the same column are significantly different (*p* < 0.05). (n = 15). GE, ginseng extract; TT, *Tribulus terrestris* extract; DPPG, date palm pollen grains.

## Data Availability

The data that support the findings of this study are available from the authors upon reasonable request.
